# Evaluating a combined bowel preparation for small-bowel capsule endoscopy: a prospective randomized–controlled study

**DOI:** 10.1093/gastro/goz054

**Published:** 2019-10-19

**Authors:** Stephanie L Hansel, Joseph A Murray, Jeffrey A Alexander, David H Bruining, Mark V Larson, Thomas F Mangan, Ross A Dierkhising, Ann E Almazar, Elizabeth Rajan

**Affiliations:** 1 Division of Gastroenterology and Hepatology, Mayo Clinic, Rochester, MN, USA; 2 Division of Biomedical Statistics and Informatics, Mayo Clinic, Rochester, MN, USA

**Keywords:** bowel preparation, capsule endoscopy, small-bowel visualization, patient satisfaction

## Abstract

**Background:**

Capsule endoscopy (CE) is frequently hindered by intra-luminal debris. Our aim was to determine whether a combination bowel preparation would improve small-bowel visualization, diagnostic yield, and the completion rate of CE.

**Methods:**

Single-blind, prospective randomized–controlled study of outpatients scheduled for CE. Bowel-preparation subjects ingested 2 L of polyethylene glycol solution the night prior to CE, 5 mL simethicone and 5 mg metoclopramide 20 minutes prior to CE and laid in the right lateral position 30 minutes after swallowing CE. Controls had no solid food after 7 p.m. the night prior to CE and no liquids 4 hours prior to CE. Participants completed a satisfaction survey. Capsule readers completed a small-bowel-visualization assessment.

**Results:**

Fifty patients were prospectively enrolled (56% female) with a median age of 54.4 years and 44 completed the study (23 patients in the control group and 21 in the preparation group). There was no significant difference between groups on quartile-based small-bowel visualization (all *P *>* *0.05). There was no significant difference between groups in diagnostic yield (*P *=* *0.69), mean gastric (*P *=* *0.10) or small-bowel transit time (*P *=* *0.89). The small-bowel completion rate was significantly higher in the preparation group (100% vs 78%; *P *=* *0.02). Bowel-preparation subjects reported significantly more discomfort than controls (62% vs 17%; *P *=* *0.01).

**Conclusions:**

Combined bowel preparation did not improve small-bowel visualization but did significantly increase patient discomfort. The CE completion rate improved in the preparation group but the diagnostic yield was unaffected. Based on our findings, a bowel preparation prior to CE does not appear to improve CE performance and results in decreased patient satisfaction (ClinicalTrials.gov, No. NCT01243736).

## Introduction

Capsule endoscopy (CE) is a diagnostic tool for investigating small-bowel lesions including sources of small-bowel bleeding. The overall diagnostic yield of CE for lesions causing small-bowel bleeding ranges from 35% to 83% [1] and the completion rate (cecal intubation) is approximately 80% [2]. The diagnostic yield or clinical usefulness of CE can be hindered by several factors including the presence of dark intestinal fluid and/or air bubbles, which limit visualization of the mucosa and thus any abnormalities within the small bowel [3].

Although the use of a bowel preparation for CE is common practice, the benefits of such an approach are not clearly addressed. Society guidelines do not offer strong recommendations in favor of purgative preparations prior to CE. Several studies have been conducted to determine whether a purgative bowel preparation, prokinetic, and simethicone improve visualization. However, the results are conflicting as to whether any bowel preparation prior to CE is helpful [4–11]. Similarly, there is controversy regarding patient positioning after capsule ingestion. Positioning may be important to decrease gastric transit time, thus improving the completion rates of CE [12, 13].

To our knowledge, there has been no prospective randomized–controlled trial investigating the use of a comprehensive combined purgative preparation with the right lateral position (RLP) to determine whether this approach will improve small-bowel visualization, diagnostic yield, and completion rates without compromising patient safety or satisfaction. Furthermore, there has been no prospective controlled trial on the use of a bowel preparation for CE in a USA-based population. Therefore, the primary aims of our study were to determine whether a combination of purgative agent, prokinetic agent, simethicone, and the RLP would improve small-bowel visualization, diagnostic yield, and the completion rate of CE. The secondary aims were safety outcomes, patient satisfaction, and gastric and small-bowel transit times.

## Materials and methods

### Study design

This was a single-blind, prospective randomized–controlled study. Subjects were block randomized equally into either the control or preparation arm of the study, and all CE readers were blinded to the study arm. All subjects gave informed written consent and the study was approved by the Institutional Review Board. The study was registered with ClinicalTrials.gov (No. NCT01243736).

### Study subjects

Consecutive patients scheduled for outpatient CE between August 2010 and July 2013 for any indication were considered for the study. Exclusion criteria included the following: (i) adverse reaction or allergy to polyethylene glycol bowel preparation, metoclopramide, or simethicone; (ii) hospitalized patients; (iii) history of a swallowing disorder; (iv) gastrointestinal motility disorder; (v) narcotic use and (vi) gastric or small-bowel resection.

### CE procedure

Control subjects were instructed to complete the standard preparation of ingesting no solid food after 7 p.m. the evening prior to the CE procedure and no liquids 4 hours prior to the CE procedure. Bowel-preparation subjects were instructed to complete 2 L of polyethylene glycol (MoviPrep^®^, Salix Pharmaceuticals, Raleigh, NC, USA) starting at 7 p.m. the evening prior to the CE procedure.

On the day of the CE procedure, all subjects were given the PillCam^®^ SB2 (Given Imaging Ltd, Israel). This device was administered by experienced endoscopic technicians in the outpatient endoscopy suite. Bowel-preparation subjects ingested simethicone 5 mL (40 mg/0.6 mL; Mylicon, McNeil Pharmaceuticals, PA, USA) and metoclopramide 5 mg (liquid preparation; Reglan, Teva Pharmaceuticals, PA) 20 minutes prior to swallowing the CE. Bowel-preparation subjects laid in the RLP for 30 minutes after swallowing the capsule. All subjects completed a questionnaire prior to leaving the procedural area.

### Outcomes

The primary outcomes of this study were small-bowel visualization, diagnostic yield, and the completion rate of CE. The secondary outcomes included safety outcomes, patient satisfaction, and gastrointestinal transit time.

CE readers completed a small-bowel-visualization four-point cleanliness scale that documented the estimated percentage of visualization in each quarter (Q) of the small-bowel transit time: Q1 (0%–25%), Q2 (26%–50%), Q3 (51%–75%), Q4 (76%–100%). Visualization was graded based on the percentage of viewable mucosa. CE readers also were required to report findings, gastric transit time, and small-bowel transit time.

A face valid satisfaction survey was given to all subjects that included any discomfort or adverse events experienced during their preparation for each specific agent and patient willingness to repeat the bowel preparation in the future. The adverse events were classified as severe if it required urgent surgery or endoscopy, or seriously threatening a patient’s health; moderately severe if it required non-urgent surgery or endoscopy, or any hospitalization (not meeting criteria for severe); and all other adverse events were classified as mild.

### Statistical analysis

The primary endpoints of small-bowel-visualization quality, diagnostic yield, and the completion rate were compared between groups using Pearson’s chi-square test. Twenty-five patients per group allowed us to detect the absolute differences of 30%–36% in the percentage with at least a fair small-bowel-visualization quartile score with 80% power (two-sided, alpha = 0.05), assuming the percentage in the control group ranged from 50% to 65%. The groups were also compared for the secondary endpoints of adverse events and patient satisfaction with Pearson’s chi-square test. A group comparison for the secondary outcomes of gastric transit time and small-bowel transit time were done using the Wilcoxon rank-sum test.

## Results

Fifty patients were enrolled (28 females; 56%) with a median age of 54.4 years (23–89 years). After enrollment, six patients withdrew from the study due to CE-procedure cancellation by the primary physician. Of the 44 patients completing the study, there were 23 patients in the control arm and 21 patients in the preparation arm. The median body mass index was 29 kg/m^2^ (range 17.5–44.1 kg/m^2^). The most common indications for CE were anemia (32%) and obscure overt gastrointestinal bleeding (32%) followed by celiac sprue (11%). Indications for the remaining 25% of CE studies included chronic diarrhea, Crohn’s disease, protein losing, autoimmune enteropathy, small-bowel mass, and abdominal pain.

### Primary outcomes

The small-bowel completion rate was significantly higher in the preparation group than the control group (21 [100%] vs 18 [78%]; *P *=* *0.02). There was no significant difference between the two groups on quartile-based small-bowel-visualization scores (all *P *>* *0.05; Table 1). Poor quartile visualizations were due to presence of dark fluid and bubbles. There was no significant difference in the diagnostic yield between the groups (preparation 38% vs control 44%; *P *=* *0.69). Clinically relevant findings on CE included small-bowel ulcers or erosions (31%), mucosal changes of celiac disease (31%), angioectasia (25%), blood without obvious lesion (6%), and small-bowel mass (6%).

**Table 1.** Small-bowel visualization by quartile among patients who completed the capsule endoscopy study

Visualization quality	No preparation (*n* = 18)	Preparation (*n* = 21)	*P*-value
1st quartile (proximal jejunum)			0.66
Poor	1 (6%)	1 (5%)	
Fair	1 (6%)	3 (14%)	
Good	6 (33%)	4 (19%)	
Excellent	10 (56%)	13 (62%)	
2nd quartile (distal jejunum)			0.38
Poor	0	0	
Fair	3 (17%)	4 (19%)	
Good	7 (39%)	4 (19%)	
Excellent	8 (44%)	13 (62%)	
3rd quartile (proximal ileum)			0.82
Poor	2 (11%)	1 (5%)	
Fair	5 (28%)	6 (29%)	
Good	2 (11%)	4 (19%)	
Excellent	9 (50%)	10 (48%)	
4th quartile (distal ileum)			0.77
Poor	4 (22%)	6 (29%)	
Fair	4 (22%)	3 (14%)	
Good	5 (28%)	4 (19%)	
Excellent	5 (28%)	8 (38%)	

Poor: <50% images showed adequate visualization of mucosa.

Fair: 50%–75% of images showed adequate visualization of mucosa.

Good: >75%–90% of images showed adequate visualization of mucosa.

Excellent: >90% of images showed adequate visualization of mucosa.

### Secondary outcomes

There was no significant difference between the preparation and control groups in gastric transit time or small-bowel transit time (both *P *>* *0.05; Table 2). The adverse-event rates seemed to differ between the groups (*P *=* *0.06). Adverse events reported by patients in the preparation group were mild and included nausea, nausea with vomiting, and inability to tolerate the entire preparation volume. Neither group reported any serious adverse events.

**Table 2.** Comparisons of secondary outcomes between groups

Outcome	No preparation (*n* = 23)	Preparation (*n* = 21)	*P*-value
Gastric transit time, minutes, median (range)	18 (12–93)	16 (8–20)	0.10
Small-bowel transit time, minutes, median (range)	215 (179–335)	244 (156–264)	0.89
Patient discomfort, *n* (%)	4 (17%)	13 (62%)	0.01
Adverse event, *n* (%)	0	3 (14%)	0.06

The patient questionnaire showed that the significant difference between the groups was discomfort (*P *=* *0.01; Table 2). The discomfort was related to the polyethylene glycol (PEG) component of the bowel preparation with the results as follows: mild discomfort 19%, moderate discomfort 24%, and severe discomfort 19%. There was little discomfort reported for simethicone (90% no discomfort), metoclopramide (86% no discomfort), or RLP (100% no discomfort) components of the bowel preparation. Despite their discomfort, the majority (76%) of the bowel-preparation subjects would be willing to undertake the same bowel preparation again in the future.

## Discussion

The purpose of our study was to determine whether the use of a unique, comprehensive combination of agents previously published to be significantly superior to no bowel preparation would improve CE performance. The combined bowel preparation of a purgative agent, prokinetic agent, simethicone, and RLP did not significantly improve small-bowel visualization compared to fasting. Moreover, the use of a bowel preparation significantly increased patient discomfort. Mild adverse events were reported in the preparation group only. The combined bowel preparation did improve the small-bowel completion rate but there was no difference in diagnostic yield. While meta-analyses did suggest bowel preparation was effective in providing better visualization and diagnostic yield [3, 14, 15], more recent individual studies continue to reveal mixed results regarding small-bowel visualization and diagnostic yield between patients with and without a bowel preparation [16–22]. Furthermore, the ideal dose and timing of purgatives before CE have yet to be determined. Our study findings are supported by recent published studies, in particular a randomized–controlled trial by Hookey *et al.* [23] that demonstrated no benefit in overall or distal small-bowel visualization with active preparation using either PEG or sodium picosulfate compared with clear fluids only.

Studies that have shown significant benefit from a bowel preparation include a Japanese study of 59 patients using 500 mL of PEG solution with significant improvement in image quality (*P *<* *0.01) and completion rate (*P *=* *0.038) [2]. Viazis *et al*. [5] compared 2 L of PEG taken 16 hours prior to CE to clear liquids only, which showed improved visualization (90% preparation vs 60% non-preparation group; *P *=* *0.004) and improved diagnostic yield (65% preparation vs 30% non-preparation group; *P *=* *0.003). A further study comparing a 12-hour fast to a 12-hour fast plus 80 mg simethicone found improved visualization (70% simethicone vs 20% non-simethicone group; *P *<* *0.01) [9].

However, other studies have found no significant improvement with the addition of a prokinetic agent or bowel preparation. A study comparing a prokinetic agent, lubiprostone 24 μg vs placebo 30 minutes prior to CE found increased gastric transit time in the lubiprostone group (126 minutes vs 43 minutes; *P *=* *0.009) but no improvement in small-bowel visualization [8]. A further study compared a clear liquid diet plus 8-hour fast to 45 mL sodium phosphate preparation and found no significant differences in small-bowel visualization, gastric transit and small-bowel transit time, or diagnostic yield [6]. Similarly, a study conducted in England with 150 patients compared four bowel preparations and found no differences in gastric transit times, completion rates, small-bowel visualization, or diagnostic yield [11].

Only a few studies have investigated whether laying the patient in the RLP improves completion rates and the results are mixed. A study of 60 patients compared laying in the RLP until the CE passed through the pylorus vs sitting upright and found the RLP reduced gastric transit time significantly (32 minutes vs 58 minutes; *P *=* *0.007) and improved the completion rate (97% vs 73%; *P *=* *0.03) [12]. However, an opposing study comparing the RLP post CE ingestion to controls found no significant difference in the gastric transit time or completion rate [13]. Notably absent from the literature are prospective controlled trials on bowel preparations for CE in USA-based populations.

Surprisingly, despite the discomfort experienced, the majority of the current study patients were willing to undertake the same bowel preparation again. Conversely, Postgate *et al*. [11] concluded that purgative agents were less convenient for patients, with a decreased acceptance of repeat CE when a preparation was used, although there are no other studies looking at this question.

The major limitation of our study was the sample size. Notably, patients eligible for the study declined participation due to concern of being randomized to the bowel-preparation group. Future studies of a combined bowel preparation with a larger sample size would be beneficial but patient willingness to participate and discomfort with bowel preparation are factors that need to be strongly considered Our small-bowel-visualization four-point cleanliness scale was a subjective assessment, but a consensus scale or objective and reliable measures for assessing the quality of bowel preparations and the definition of adequate visualization for CE are lacking. In addition, we excluded patients who may be more likely to have poor visualization or incomplete exams, such as hospitalized patients and those on narcotics.

In conclusion, a combined bowel preparation of a purgative agent, prokinetic agent, simethicone, and the RLP did not significantly improve small-bowel visualization but did significantly increase patient discomfort with mild adverse events. The completion rate of CE improved in the bowel-preparation group but the diagnostic yield was unaffected. It is our practice not to perform any preparation for CE except an overnight fast, which is supported by the study findings and other published data. Rather than routinely prescribing bowel preparations for CE, we should identify select patient populations who would benefit from this approach. Based on our findings, a bowel preparation prior to CE does not appear to improve CE performance and results in increased patient discomfort.

## Authors’ contributions

Conception and design: S.L.H. and E.R. Analysis and interpretation of data: S.L.H., R.A.D., A.E.A., and E.R. Drafting of article: S.L.H. Critical revision of article: all authors. Final approval of the article: S.L.H. and E.R.

## Funding

Salix Pharmaceuticals, Raleigh, NC, USA.
